# The Risks and Outcomes Resulting From Medication Errors Reported in the Finnish Tertiary Care Units: A Cross-Sectional Retrospective Register Study

**DOI:** 10.3389/fphar.2019.01571

**Published:** 2020-01-17

**Authors:** Outi Laatikainen, Sami Sneck, Miia Turpeinen

**Affiliations:** ^1^Research Unit of Biomedicine, Department of Pharmacology and Toxicology, and Medical Research Center Oulu, University of Oulu, Oulu, Finland; ^2^Administration Center, Oulu University Hospital, Oulu, Finland

**Keywords:** medication error, medication safety, patient safety, tertiary care, pharmaceutical care

## Abstract

**Background:** Hospital-acquired medication errors (MEs) are common in health care. Although voluntary reporting is criticized for not producing reliable estimates on ME frequency, it provides valuable knowledge on errors occurring in the medication process.

**Objective:** The purpose of this study was to analyze and determine the risks and outcomes resulting from MEs related to the TOP15 medicines in the Finnish tertiary care units from July 2016 to July 2017.

**Methods:** The data consisting of 1,447 ME reports was organized according to ATC classification, after which TOP15 medicines involved in the reports were selected. Inductive content analysis was performed to the reports. After this, the reports were categorized by ME outcome into five categories and further analyzed accordingly.

**Results:** The most common ME outcome in the reports was “omitted medicine” (33.9%). More than a quarter (27.1%) of ME reports were estimated to cause moderate or severe risk to the patient. When compared with each other, none of the outcome groups were more susceptible to high-risk events (p = 0.71). Of the TOP15 medicines, only Norepinephrine had significantly higher risk of being involved in high-risk events (OR 2.43, 95%CI 1.35–4.61).

**Conclusion:** Voluntary reporting has an important role in the development of medication safety and the overall medication process within organizations. Although majority of the TOP15 medicines were involved in MEs resulting in seemingly high-risk outcomes, they were estimated to be insignificant or minor within the reporting unit. In the future, more emphasis will be needed for the assessment and analysis of the reports for more efficient, real-time detection and response to signals from health care units.

## Introduction

Medication errors (MEs) are highly common in an in-hospital setting (Kaushal et al., 2001; Rothschild et al., 2007; Aronson, 2009; Carayon et al., 2014; Aibar, et al., 2015; Härkänen et al., 2018). They can occur at any stage of medication process, from prescribing to handling and administering. Approximately 50% of MEs result in adverse drug events (ADEs) causing significant increase in patient morbidity and mortality as well as in economic costs in health care (Hohl et al., 2001; Krähenbühl-Melcher et al., 2007; Alhawassi et al., 2014; Classen et al., 2016; Walsh et al., 2017). As ADEs caused by MEs are generally considered preventable, understanding MEs is particularly important when developing and improving preventative methods towards medication-related patient harm (Morimoto et al., 2004; Asaad Assiri et al., 2018; Wang-Hansen et al., 2019).

As the awareness of MEs has grown, different detection methods have been developed to monitor the events. Voluntary reporting systems, where the reports are typically drawn up by health care professionals, are one of the most commonly used methods (Gandhi et al., 2000; Montesi and Lechi, 2009; Choi et al., 2016). Although voluntary reporting is not the best method for providing reliable estimates of the prevalence of MEs, it provides low-cost means to describe errors occurring in the medication process and is therefore widely used in various care settings (Choi et al., 2016).

In Finland, nationwide voluntary reporting system (Haipro) for patient safety incident reports was developed in 2007, and quickly after piloting expanded to cover the majority of all public health care units. Currently, it is used by over 200 units in health care and social services in Finland. The Haipro system consists of patient safety incident reports from different fields of medical care, thus also including medication safety incident reports. The reports mainly consist of MEs and near miss events, although some direct ADEs can also be reported. The events are reported by health care professionals and patients. Annually, the Haipro-system produces approximately 15,000 medication safety incident reports nationwide, creating the largest database for ME reports currently available in Finland.

Although the medication safety incident reports have now been collected for more than 12 years, the conclusive, in-depth analysis of the national reports is still lacking. The main objective of this study was to describe the types and outcomes of MEs related to the medicines most commonly involved in medication safety incident reports and to abstract the medicine-specific risk associated with these medicines.

## Materials and Methods

### Study Setting

This was a cross-sectional retrospective register study on tertiary care (university and central hospital level specialized care) MEs. The data consisted of 9,269 medication safety incident reports (Haipros) collected from the Finnish tertiary care units from July 2016 to July 2017. The data included 77% of all reports made in Finland during the data collection period, excluding only the reports of one of the five hospital districts in Finland as they denied access to their reports. However, as the data included the reports of four out of five hospital districts, the data represents well the current situation in Finland. Only the reports describing MEs were included in this study. All data used in the study was anonymized. Here, a ME was defined as a preventable error which, when left undetected, may lead to inappropriate medication use and harm to the patient (Aronson, 2009; Becker, 2015). The research was conducted according to Standards for Reporting Qualitative Research (SRQR).

The collected medication safety incident reports consisted of two types of information: categorical and narrative. The categorical information can easily be converted into quantitative data whereas narrative information requires analyzing and quantifying before any conclusions can be derived. In the reports, categorical information included a description of e.g. event category, risk, and patient outcome assessed by the unit supervisor within the reporting organization. The risk assessment in the reports was conducted using a special matrix designed for this purpose. The matrix was the same for all organizations using the Haipro system. The definitions for the risk categories used in the reports are presented with examples in **Appendix 1** (Giardina et al., 2018).

Narrative information in the reports included the description of the medicines involved in the events and the event itself. In this study, inductive content analysis methods were used to analyze and extract the narrative information in the medication safety incident reports. The extracted data was then combined with the categorical risk assessment in the reports.

### Data Processing

**Figure 1:**
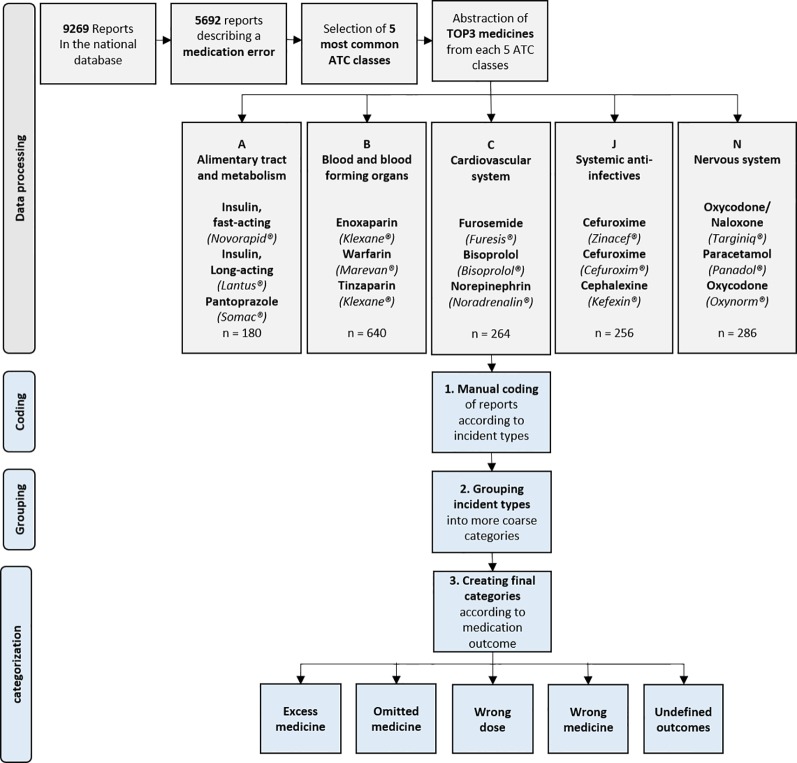
Study design and outline of inductive content analysis.

The data was first screened for identifying the reports of MEs only, after which the selected reports were imported to QSR Nvivo (^©^QSR Nvivo International Pty Ltd) for processing (**Figure 1**). In QSR Nvivo, all reports were categorized into ATC groups according to medicines involved in them, using search terms created out of every brand name and active substance name currently on the Finnish market. After categorization, five ATC groups with the highest number of medication safety incident reports were selected for further processing. The selection was endorsed by the previous results on in-hospital MEs and ADEs (Giardina et al., 2018; Wolfe et al., 2018).

After this, the TOP3 medicines from the selected five ATC groups were extracted for inclusion in this study. This extraction was conducted by searching the terms most frequently used in the reports, either as an active substance name or a brand name. This approach was adopted due to the fact that significant proportion of medication errors include events which can be specific for the brand name or appearance of the used medicinal product (Look alike, sound alike -errors) (Emmerton and Rizk, 2012; Larmené-Beld KH et al., 2018). Finally, the included reports were grouped together for the final inclusion of the TOP15 medicines involved in MEs during 2017. After this, the data was further categorized and analyzed using inductive content analysis methods.

### Inductive Content Analysis

All reports including TOP15 medicines were carefully read through in QSR Nvivo. In the first phase, each report was analyzed according to incident type and assigned a specific code describing the incident with high accuracy, e.g. “patient received other patients' medicine” (**Figure 1**). In the second phase, codes describing the events were reanalyzed to detect different error types in the codes. According to this analysis, the codes were listed roughly in groups, e.g. “Wrong active substance given to the patient”. In the third phase, the groups were again analyzed for detection of any themes or categories between the formed groups. The groups were then further abstracted into categories describing the medicinal outcome caused by the incident in a more general level, e.g. “wrong medicine”. The created categories for medicinal outcome were incorporated with other existing data and used in further quantitative analysis. However, for the groups “wrong medicine” and “wrong dose” the subgroups “too high dose”, “too low dose”, “wrong active substance”, and “wrong formulation” were also included in the analysis whenever possible.

In this study, the omission of medicine was defined as patient not receiving the dose of medicine intended in the prescription of medication regimen. Prescribing error was defined as an error occurring in physician’s prescriptions during the admission, and transcribing error as an error in the transferring of prescription (verbal or written) to the electronic patient files by a non-prescribing staff member, e.g. a nurse or a pharmacist. Dispensing errors were errors that occurred in the dispensing of any drug in the patient's medication regimen during hospital stay. Random errors were any mishaps resulting in any type of erroneous event in the execution of pharmacological treatment during admissions, while LASA (Look Alike, Sound Alike) errors were errors that occur due to similar appearance or similar brand name or active substance name of two different medicines.

### Statistical Analysis

After qualitative analysis, all data was imported to SPSS Statistics 25.0 (SPSS Inc., Chicago, IL, USA) for further analysis. Descriptive statistics were used to describe the amounts and distribution of certain variables in the data. Pearson's chi-squared (χ^2^) test was used to test the relationship between discontinuous variables in the reports, e.g. assessed risk, medicinal outcome, and medicines involved. Odds ratio (OR) was used to assess the association of medicinal outcomes, risk categories, and TOP15 medicines. P value 0.05 was selected as level of statistical significance in the two-sided approach.

Due to the nature of the data, there was partial overlap between the reports in the variables ME outcome and medicines involved. To minimize the bias caused by this, the analyses of medicine-specific risk and medicinal outcome was conducted with only the reports that included one TOP15 medicine. In total, 168 reports including 347 medicines were excluded from the analysis.

## Results

There were 9,269 medication safety incident reports made in the Finnish tertiary care units from July 2016 to July 2017. Of these reports, 5,692 (61.4%) concerned MEs and were selected for this study. When the TOP3 medicines from the five most commonly involved ATC groups (A, B, C, J, and N) were selected, a sample of 1,447 reports were included in the final analysis. In the included 1,447 reports, 1,626 medicines were identified. Of these medicines, insulins (long-acting Lantus^®^, fast-acting Novorapid^®^) covered 8.2%, intravenous anti-infectives (Cefuroxime^®^, Zinacef^®^) 13.2%, opioids (Targiniq^®^, Oxynorm^®^) 11.7%, oral anticoagulants (Marevan^®^) 10.4%, and low-molecular weight heparins (Klexane^®^, Innohep^®^) 33.8%. Multiple TOP15 medicines were involved in 168 (11.6%) of the included reports (**Table 1**). Warfarin (Marevan^®^, 41.4%) was the medicine most commonly used in association with other drugs whereas Norepinephrine (Noradrenalin^®^, 9.4%) was the least.

**Table 1:** Associations of the TOP15 medicines in the medication safety incident reports.

	Novorapid^®^	Lantus^®^	Somac^®^	Klexane^®^	Marevan^®^	Innohep^®^	Furesis^®^	Bisoprolol^®^	Noradrenalin^®^	Zinacef^®^	Cefuroxim^®^	Kefexin^®^	Oxynorm^®^	Panadol^®^	Targiniq^®^
**Novorapid**^®^	**76**	12	1	0	2	0	2	0	2	2	0	0	0	0	0
**Lantus**^®^	12	**57**	0	1	1	0	2	1	0	1	0	0	0	0	0
**Somac**^®^	1	0	**47**	3	2	0	6	2	0	2	0	0	0	1	1
**Klexane**^®^	0	1	3	**380**	43	8	7	4	0	2	0	0	1	1	1
**Marevan**^®^	2	1	2	43	**169**	9	3	4	1	3	0	1	0	0	1
**Innohep**^®^	0	0	0	8	9	**91**	1	0	0	0	0	0	0	0	1
**Furesis**^®^	2	2	6	7	3	1	**147**	8	1	2	0	0	0	3	2
**Bisoprolol**^®^	0	1	2	4	4	0	8	**64**	0	0	0	0	0	0	1
**Noradrenalin**^®^	2	0	0	0	1	0	1	0	**53**	1	0	0	0	0	0
**Zinacef**^®^	2	1	2	2	3	0	2	0	1	**153**	5	5	1	1	0
**Cefuroxim**^®^	0	0	0	0	0	0	0	0	0	5	**62**	4	0	0	0
**Kefexin**^®^	0	0	0	0	1	0	0	0	0	5	4	**41**	0	1	1
**Oxynorm**^®^	0	0	0	1	0	0	0	0	0	1	0	0	**83**	6	11
**Panadol**^®^	0	0	1	1	0	0	3	0	0	1	0	1	6	**95**	5
**Targiniq**^®^	0	0	1	1	1	1	2	1	0	0	0	1	11	5	**108**
**% distribution**	27.6%	31.6%	38.3%	18.7%	41.4%	20.9%	25.2%	31.3%	9.4%	16.3%	14.5%	29.3%	22.9%	18.9%	22.2%

The overall number of reports per medication marked with bolded numbers.

**Figure 2:**
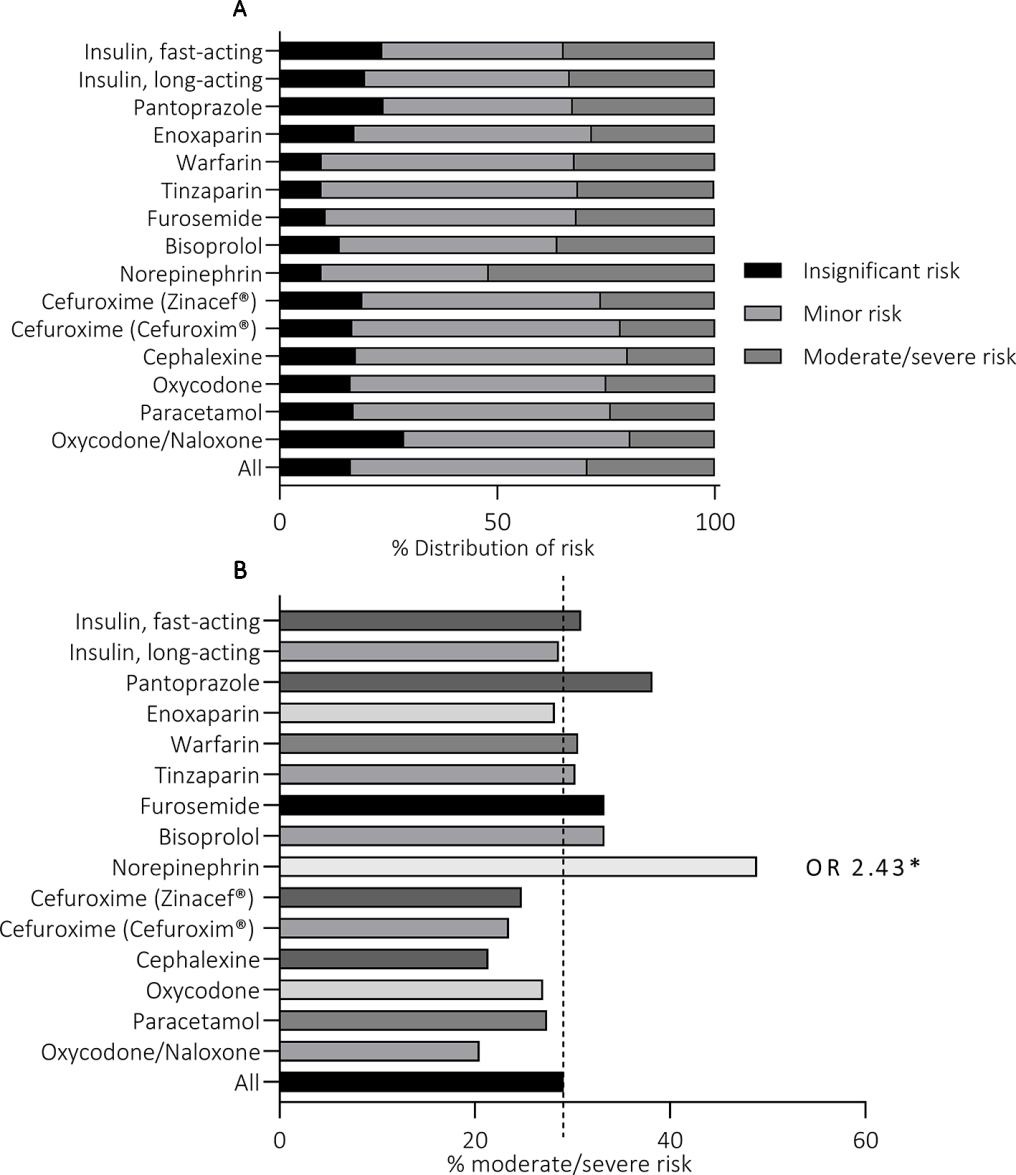
Medication error outcomes of 1,447 medication safety incident reports **(A)** and the risk associated with each outcome **(B)**. *p < 0.05.

In the reports, a total of 1,483 different MEs were detected. All MEs were categorized into five main groups according to the ME outcome. The majority of reported events (n = 509, 33.9%) resulted in “omitted medicine” outcome (**Figure 2**). The rest of the reports were divided into the remaining 4 outcomes with ourvariation from 14.9% to 19.4% per group. In the “wrong dose” group, 68.8% (n = 163) of the reported incidents were administration of a too high dose and 32.2% (n = 76) of a too low dose. Furthermore, in the group “wrong drug”, 79% (n = 177) of the cases reported administration of wrong active substance and 21% (n = 47) administration of wrong formulation. MEs were categorized as “undefined outcomes” when the outcome of the event remained unclear. Such reports included e.g. administration of medicine *via* wrong administration route, administration of Warfarin (Marevan^®^) without up-to-date prescription and dosage, formation of precipitate during intravenous administration, and administration of unsuitable medicines (e.g. allergies). Undefined outcomes covered 19.4% (n = 291) of the reports.

The majority (72.9%) of MEs in all outcome groups were assessed to cause insignificant or minor risk to the patient (**Figure 2**). The overall number of MEs causing moderate risk was 373 (25.8%), with percentage per outcome group varying from 22.6% (“excess medicine”) to 26.8% (“wrong medicine”). Similarly, the total number of MEs causing severe risk was 19 (1.3%), with lowest incidence in “wrong dose” (0.4%, n = 1) and highest in “wrong medicine” (2.7%, n = 6) outcome groups. Statistically significant variation was not detected in the distribution of high-risk events between the outcome groups (p = 0.71).

**Figure 3:**
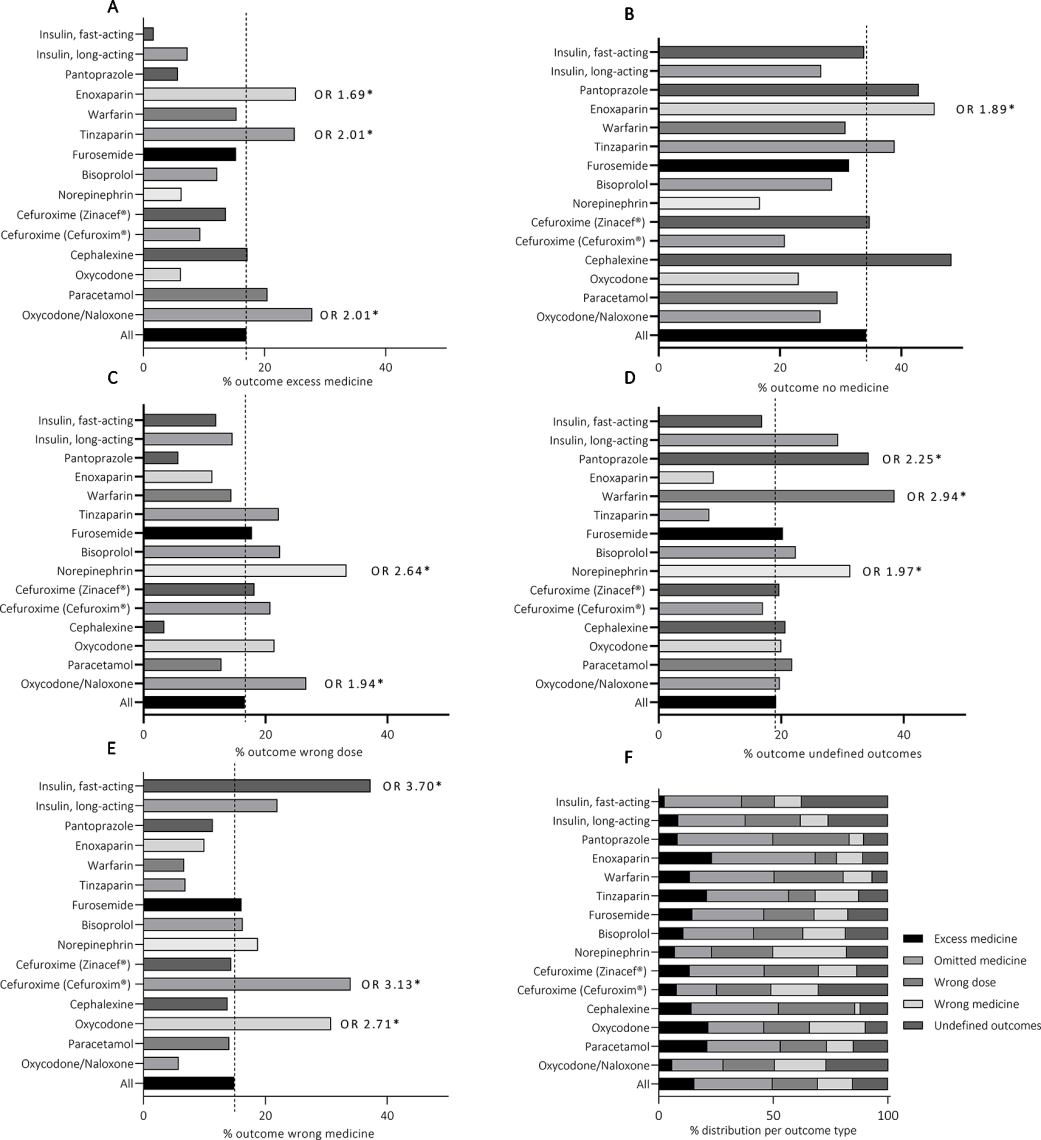
Medicine-specific medication error outcomes **(F)** and the distribution of each outcome within the TOP15 medicines **(A**–**E)**. *p < 0.05.

The distribution of ME subcategories of each medicinal outcome is presented in **Table 2**. A ME resulted in the outcome “omitted medicine” most frequently due to a random error, typically a simple mishap in the administration process, and errors in transcribing or interpreting prescriptions. Compared with other medicines, Enoxaparin (Klexane^®^), had significantly higher risk for this ME outcome (OR 1.89, 965% CI 1.45–2.45) (**Figure 3**). Random error and errors in transcribing and interpreting prescription were also the most common ME subcategories in the outcomes “too high dose” and “too low dose”. Norepinephrine (Noradrenalin^®^, OR 2.64, 95% CI 1.42–4.90) and Oxycodone/Naloxone (Targiniq^®^ OR 1.94, 95% CI 1.17–3.20) had significantly increased susceptibility to the outcome “wrong dose”. The MEs most frequently resulting in “excess medicine” were random errors, errors in information transfer in care interface, and errors with printed medication lists. Transfer of medication information in care interface, i.e. between different units, care facilities or staff shifts, was also the most common cause of errors resulting in “undefined outcome”. Enoxaparin (Klexane^®^, OR 2.01, 95% CI 1.47–2.75), Oxycodone/Naloxone (Targiniq^®^ OR 1.69, 95% CI 0.98–2.94), and Tinzaparin (Innohep^®^, OR 2.01, 95% CI 1.22–3.29) were significantly more susceptible to the outcome “Excess medicine” whereas Pantoprazole (Somac^®^, OR 2.25, 95% CI 1.11–4.59), Warfarin (Marevan^®^, OR 2.94 95%CI 1.93–4.49), and Norepinephrine (Noradrenalin^®^, OR 1.97, 95%CI 1.05–3.68) typically resulted in “undefined outcome”. Finally, the administration of wrong insulin and administration of medicine to a wrong patient were the most frequent subcategories in the outcome “wrong medicine”. Fast-acting insulin (Novorapid^®^, OR 3.70, 95% CI 2.13–6.42), Cefuroxime (Cefuroxim^®^, OR 3.10, 95% CI 1.73–5.65), and Oxycodone (Oxynorm^®^, OR 2.71, 95% CI 1.56–4.70) were found having significantly higher risk for this type of events. Moreover, the odds ratio of the fast-acting insulin (Novorapid^®^) for being given to a patient whose medication regimen did not include fast-acting insulin at all was the highest of all medicines in any outcome group. Moreover, in the case of Cefuroxime (Cefuroxim^®^) the events were evidently caused by mix-ups with antibiotics with similar names, such as ceftriaxone or ceftazidime. Similar errors were not present with the other Cefuroxime preparation (Zinacef^®^), indicating an increased potential for LASA errors with this particular preparation.

**Table 2:** Medication error outcomes and subcategories created in the inductive content analysis.

	Excess Medicine n = 239	Omitted Medicine n = 502	Wrong Dose		Wrong Medicine		Undefined Outcomes n = 286
			Too High Dose n = 163	Too Low Dose n = 76	Active Substance n = 177	Formulation n = 47	
**Subcategories**	Random error (26.0%) Transferring information in care interface (24.0%) Errors with printed lists (23.1%) Transcribing or interpreting prescription (15.3%) Prescribing errors (4.1%) Errors in documenting administration (3.7%) Technical errors in administration (1.2%) LASA errors (1.2%) Others (1.2%)	Random error (35.3%) Transcribing or interpreting prescription (20.6%) Errors with printed lists (19.4%) Transferring information in care interface (11.1%) Others (5.2%) Prescribing errors (4.3%) Technical errors in administration (3.3%) Errors in documenting administration (0.8%)	Random error (39.3%) Transcribing or interpreting prescription (22.1%) Others (14.7%) Transferring information in care interface (6.7%) Prescribing errors (5.5%) Errors with printed lists (5.5%) Calculating doses or concentrations (3.7%) LASA errors (2.5%)	Transcribing or interpreting prescription (35.1%) Random error (24.7%) Technical errors in administration (11.6%) Transferring information in care interface (10.4%) Prescribing errors (5.2%) Errors with printed lists (5.2%) Calculating concentration (3.9%) Errors in generic substitution (2.6%) LASA errors (1.3%)	Administering medicine to wrong patient (65.4%) LASA errors (15.6%) Transferring information in care interface (6.6%) Prescribing errors (4.4%) Transcribing or interpreting prescription (3.8%) Errors with printed lists (2.8%) Others (1.4%)	Administration of wrong insulin (57.4%) LASA errors (19.1%) Transcribing or interpreting prescription (8.5%) Others (8.5%) Prescribing errors (4.3%) Errors with printed lists (2.1%)	Transferring information in care interface (35.2%) Prescribing errors (13.7%) Errors in patient files (10.9%) Administration of unsuitable medicine (10.2%) Technical errors in administration (8.9%) Non-rational treatment (7.5%) Using expired medicine /mishandling medicine (6.5%) Not defined (4.2%) LASA errors (1.7%) ADR (1.3%)

**Figure 4:**
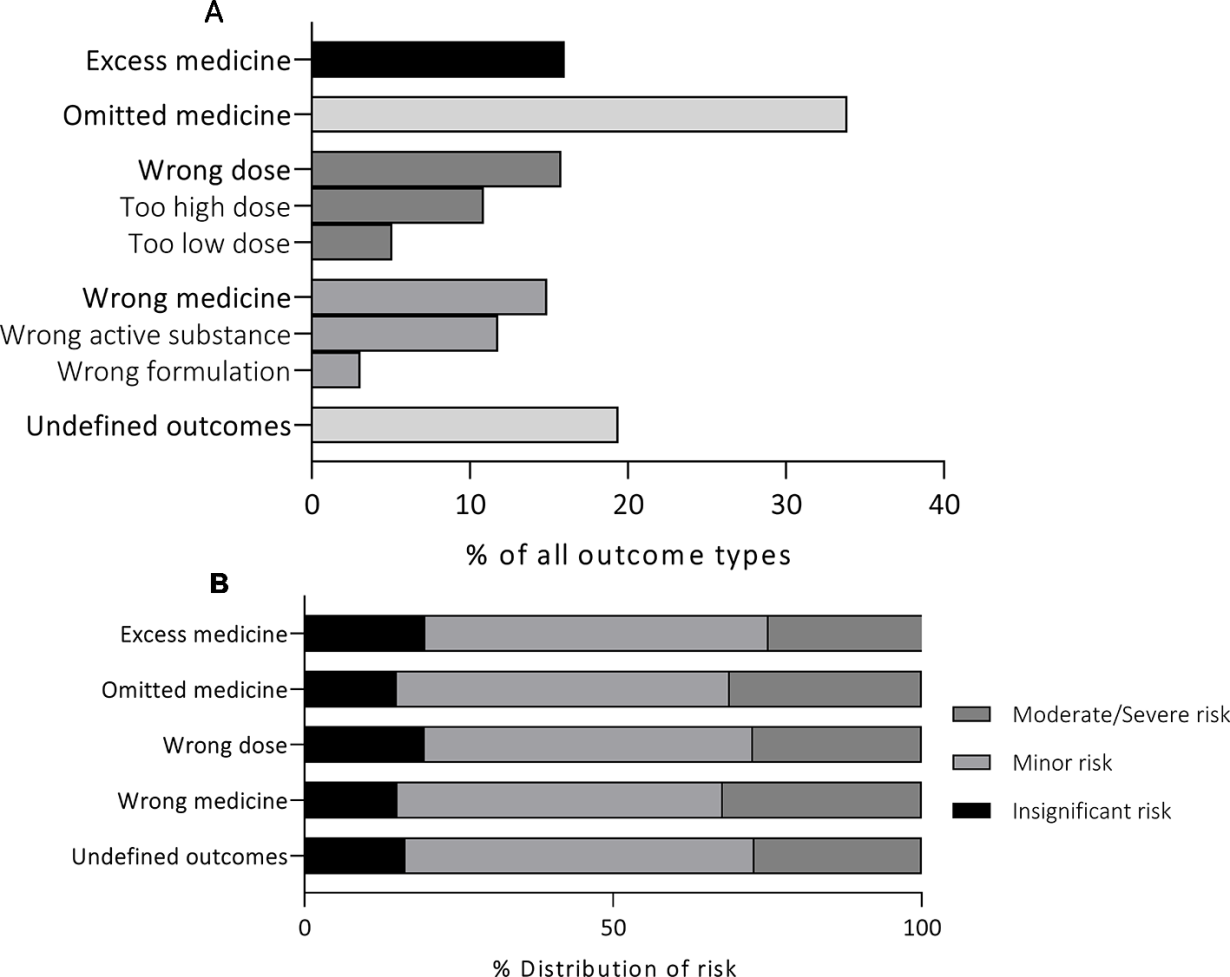
The distribution of the assessed risk within the TOP15 medicines **(A)** and the high risk events related to each of the TOP15 medicines **(B)**.

Statistically significant variation was detected between the TOP15 medicines in the proportion of high-risk events (p = 0.04) (**Figure 4**). Noradrenalin^®^ was the only medicine with significantly higher frequency for incidents that were assessed to cause moderate or severe risk to the patient (OR 2.43, 95%CI 1.35–4.61). The majority of the reported events involving Norepinephrine were administration of expired medicines (“undefined outcomes”), administration of wrong medicine due to LASA errors (“wrong medicine”), administration of a wrong dose due to technical issues, and administration of medicine *via* wrong administration route (“undefined outcomes”). Increased odds ratios were detected with other TOP15 medicines as well, but the results were not statistically significant (p > 0.05) and were thus disregarded.

## Discussion

In this study, MEs were analyzed using the Finnish national medication safety incident report data from tertiary care units in 2017. Consistent with former research, the medicines most commonly involved in the reports were from ATC groups A (Alimentary tract and metabolism), B (Blood and blood-forming organs), C (Cardiovascular system), J (Anti-infectives for systemic use), and N (nervous system) (Laatikainen et al., 2017; Giardina et al., 2018). Furthermore, the majority of the TOP15 medicines were preparations that are considered high-risk medicines in international listings, i.e. anticoagulants, insulins, adrenergic agonists/antagonists, and opioids (Institute for Safe Medication Practices, 2018). As with former research, in this study administration errors also occurred slightly more frequently with parenteral products than with perorally administered preparations (Keers et al., 2013). The results highlight the fact that, alongside pharmacology, the high-risk nature of medicines is also largely dependent on their use and susceptibility to process-based errors: even the safest medicine can cause significant harm if it is not used appropriately. Thus, close surveillance of such errors in the medication process is imperative in the development of medication safety in health care.

In this study, individual reports were analyzed for describing the ME outcome to further improve the understanding of the extent MEs have on the medical care of patients. Medicine-specific differences were discovered in the susceptibility to certain outcomes within the TOP15 medicines: both low-molecular weight heparins (Enoxaparin and Tinzaparin) had a 2-fold risk for MEs resulting in administration of excess medicine. Norepinephrine (Noradrenalin^®^) had 2 to 3-fold risk for MEs resulting in either administration of a wrong dose or undefined outcomes, and fast-acting insulin (Novorapid^®^) had almost 4-fold risk for an error resulting in the administration of wrong medicine. Furthermore, one of the intravenous Cefuroxime preparations (Cefuroxim^®^) was discovered to have significantly increased susceptibility to LASA-errors compared with another generic preparation (Zinacef^®^). Thus, the results demonstrate the possibilities with ME data. Not only are they valuable in describing the errors creating high-risk situations in the care processes but they also encompass the potential of pinpointing errors typical to certain medicines and thus facilitate the identification of high-risk medicines in current use providing great opportunities for the planning of safer processes.

The risk associated with MEs was assessed as moderate or severe in more than a quarter (27.1%, n = 319) of the reports. According to the assessment conducted within the organizations, Norepinephrine (Noradrenalin^®^) was the only one with significantly higher susceptibility to high-risk events. Although the events reported for Norepinephrine were undeniably severe, similar estimates could also be made for the incidents involving the other TOP15 medicines as well: The most common errors including fast-acting insulin (Novorapid^®^) were the administration of insulin to a wrong patient or confusing it with another medicine due to LASA-errors—both incidents that have been linked to severe consequences in the past (Classen et al., 2010; Geller et al., 2014). Furthermore, the excess administration of low-molecular weight heparins (Tinzaparin, Enoxaparin) or, on the other hand, the omitted administration of them, could also result in severe consequences. This raises questions whether accurate assessments can be achieved by processing ME reports locally within the reporting units. It is possible, that this procedure increases bias as the assessment is conducted by individuals with less pharmacological expertise. It should also be considered whether bias could be formed if the person assessing the events is familiar with the personnel and the unit practices. Although the inter-rater reliability of reported risk has not been assessed before, similar discrepancies in the inter-rater reliability of several other variables have been detected with comparable data (Holmström et al., 2018).

As a detection method, voluntary reporting is widely criticized for both its inability to produce accurate estimates of the overall prevalence of MEs as well as the apparent under-reporting related to it (Nuckols et al., 2007; Sari et al., 2007; Manias, 2013). However, it has great significance in gathering massive amounts of data on not just MEs but all medication-related adverse events in health care. Furthermore, it produces invaluable descriptions of the care stages most susceptible to errors. During the past years, increased patient numbers have also fostered an increase in ME data resulting in challenges in data processing and analysis. Thus, the utilization of the reports produced by voluntary reporting systems have become undermined by the laborious and slow analysis with apparent challenges also in the reliability and coherence of the current analysis process. For tackling these difficulties in the future, computerized methods have proven both efficient and accurate (Montesi and Lechi, 2009; Molokhia et al., 2009; Manias, 2013). Trigger tools have been shown to markedly improve event detection on their own, but with incorporation to incident reporting systems they have the possibility to cover wider range of medication-related adverse events. Accordingly, incorporation of such systems with incident reports could provide valuable improvements in both utilizing this resource and turning incident reporting from retrospective surveillance towards more active form of prevention.

Finally, there are several limitations to this study. The nature of the data, i.e. voluntary reporting, may result in bias that appears in overexpression of certain MEs and medicines as well as under-expression of others as only approximately 5–10% of overall medication-related adverse events are reported (Desikan et al., 2005; Härkänen et al., 2016). It is possible, that certain medicines and active substances were not discovered with the ATC based searches made in QSR Nvivo due to misspelling or the use of professional slang. Although high accuracy was achieved in the analysis by excluding cases involving more than 1 TOP15 medicines, some information was inevitably lost by choosing this approach. However, in Finland similar studies with national data are lacking. The results of this study are the first explicit analysis of the ME outcome in the Finnish tertiary care units and are also a good representative of the current situation in all western countries.

## Conclusions

Medicines most frequently involved in medication safety incident reports in the Finnish tertiary care units during 2017 were from ATC groups A (Alimentary tract and metabolism), B (Blood and blood forming organs), C (Cardiovascular system), J (Anti-infectives for systemic use), and N (Nervous system). The most frequent medication error outcome was “omitted medicine”. Although several medicines were linked to serious MEs during the inductive content analysis, only Norepinephrine had significantly increased susceptibility to high-risk events according to the assessment conducted within the units. In the future, improved methods are needed for the assessment and utilization of voluntary reports as they provide valuable information on process-based errors in health care organizations.

## Data Availability Statement

All datasets generated for this study are included in the article.

## Author Contributions

All authors (OL, SS, MT) contributed to the study conception and design. SS processed and organized the data. OL and SS analyzed the data. The first draft of the article was written by OL and all authors provided comments on the previous version of the manuscript. All authors read and approved the current manuscript.

## Conflict of Interest

The authors declare that the research was conducted in the absence of any commercial or financial relationships that could be construed as a potential conflict of interest.

## Appendix 1

**Table:** Definitions and Examples of the Risk Categories Presented in the Study (Giardina et al., 2018).

Risk category	Definition	Example
Insignificant	Errors that cause no significant health impacts	One-time omission of medicine resulting in delayed administration
Minor	Errors that cause minor discomfort with no significant health impacts	Omission of medicine used ´”when needed”, e.g. antacids
Moderate	Errors that cause the need for minor treatment, increase length of stay and cause temporary consequences to the patient	Administering wrong dose of a medicine occurring in patients medication regimen.
Major	Errors that cause long-term or permanent consequences to the patient, require immediate intervention to prevent further harm	Administration of wrong doses of life-supporting medicines, e.g. norepinephrine
